# A Great Matron's Personality

**Published:** 1919-02-22

**Authors:** 


### A GREAT MATRON'S PERSONALITY.

### THIRTY-NINE YEARS' MATRONSHIP.

We have thought it best to rely on the kindly co-operation of the Chairman and officers of the London Hospital for the most interesting and educative facts, some of which have an uplifting value of great price, which we publish elsewhere, embodying the ideals, work, and career of an old friend and good woman, Miss Eva Charlotte Luckes, R.R.C., C.B.E. Many times and oft have we enjoyed with her long conferences and prolonged debates upon the policy, developments, and pressing needs of the Nursing profession during the last forty years. Her keenness of perception, her courageous and continuous resistance of abuses and evils which she made it her business to fight throughout a long official life, her eager readiness to do a kindness, to help to supply aid in a multitude of critical cases, when difficulties arose, and her ever-ready sympathy, unfailing, faithful and hearty friendship during half a lifetime, make her loss very real to all who had the privilege of her confidence and friendship. She ought to have been a Yorkshirewoman, for she was in fact a genuine forceful leader, and when she had made up her mind that a certain course of action or plan of administration or system of work was *the* plan, that plan prevailed and had to endure so long as she had command of the ship. These attributes gave an interest to her whole life's work, made her fruitful as a study in character, and won for her admiration, and recognition for thoroughness from the ablest hospital administrators in the land, which will make her work live in their memory so long as life endures. Kind-hearted, generous and resolute, she possessed the power of enforcing discipline in and imparting thoroughness to the members of her staff, in all the various departments of work to be met with in a great hospital. Her rule produced on the whole most excellent results during the years when she was able to use her own eyes and ears in the affairs of the hospital, including the condition and efficiency of individual workers in each of the various departments and throughout the most important portions of a great hospital, the wards and accommodation for the treatment of patients, and the comfort and welfare of the nursing staff.

Her memory for persons and faces, for facts and events in relation to almost every detail of the affairs of the hospital to which she devoted her life, and to the history, experiences, and accomplishments of each nurse on the establishment within and without the hospital, working in distant parts of the country or abroad, or serving on the private staff, was a source of envy to many of her contemporaries who had the honour to preside over great hospitals or kindred institutions. She never seemed at fault, and a mere diary of the incidents and events which took place in her own office, arising out of the daily work which was done there during the thirty-nine years of her matronship, as may be inferred from the accounts we publish in the following pages by writers who had the advantage of working with her, learning her capacity and strength, and were devoted to her by bonds of affection, if placed in the hands of a competent writer should result in a book of permanent value and the deepest interest to the hospital world. The work which devolves upon the holders of high office in great institutions, and especially in hospitals of the voluntary type in this country, becomes more and more absorbing as knowledge and opportunity grow, the demands and claims of the institution upon each member of its staff increase, and its reputation as an exemplar and centre of the highest work, and diffuser of knowledge comes to be recognised by the nation, and those who devote their lives to the succour and uplifting of the suffering and the sick.

Miss Luckes began hospital life with definite ideals which, as we show elsewhere, endured to the end, and won for her a great position. Such a position all may aspire to reach, but few can hope to attain. She encouraged reciprocity between principals, and we have known her to spend weeks in procuring and securing the accuracy and completeness of important returns, full of value not only to her institution and departments of work, but of permanent interest too. She sent them to us in due course, and we have them carefully preserved to-day as documents of reference, authority and value.

She was prepared to give, and to give of her best, to every one whom she trusted or felt to be in a position to ask for it, and she neither forgot a promise nor failed to fulfil it in all the long period of our association and varied and interesting work together. Her memory will be treasured by her friends, carefully guarded by her associates and held in deep respect by institutional workers who possess the experience, resting on knowledge, which fits men and women for responsible posts, and gives them the power justly to rule others. Her persistent clinging to the post of duty to the very last day of her life will probably never be forgotten. Her ambition was to die in harness, and she attained this end with complete success by the exercise of great and continuous self-control through all the painful stages of her most trying and prolonged illness. All honour to her memory. May British hospitals always command the services of noble women of equal character, authority, knowledge and will-power. Then indeed may we rest assured that not only our hospitals but our nurses will prove themselves to be admittedly the best in the world.

